# Ketogenic Diet in the Treatment of Super-Refractory Status Epilepticus at a Pediatric Intensive Care Unit: A Single-Center Experience

**DOI:** 10.3389/fneur.2021.669296

**Published:** 2021-06-03

**Authors:** Markus Breu, Chiara Häfele, Sarah Glatter, Petra Trimmel-Schwahofer, Johann Golej, Christoph Male, Martha Feucht, Anastasia Dressler

**Affiliations:** Department of Pediatrics and Adolescent Medicine, Medical University Vienna, Vienna, Austria

**Keywords:** ketogenic diet, status epilepticus, pediatric, beta-hydroxybutyrate, parenteral diet

## Abstract

**Background:** To evaluate the use of the ketogenic diet (KD) for treatment of super-refractory status epilepticus (SRSE) at a pediatric intensive care unit (PICU).

**Design:** A retrospective analysis of all pediatric patients treated for SRSE with the KD at our center was performed using patient data from our prospective longitudinal KD database.

**Setting:** SRSE is defined as refractory SE that continues or recurs 24 h or more after initiation of anesthetic drugs. We describe the clinical and electroencephalographic (EEG) findings of all children treated with KD at our PICU. The KD was administered as add-on after failure of standard treatment. Response was defined as EEG seizure resolution (absence of seizures and suppression–burst ratio ≥50%).

**Patients:** Eight consecutive SRSE patients (four females) treated with KD were included. Median age at onset of SRSE was 13.6 months (IQR 0.9–105), and median age at KD initiation was 13.7 months (IQR 1.9 months to 8.9 years). Etiology was known in 6/8 (75%): genetic in 4 (50%), structural in 1 (12.5%), and autoimmune/inflammatory in 1 (12.5%).

**Main Results:** Time from onset of SRSE to initiation of KD was median 6 days (IQR 1.3–9). Time until clinically relevant ketosis (beta-hydroxybutyrate (BHB) >2 mmol/L in serum) was median 68.0 h (IQR 27.3–220.5). Higher ketosis was achieved when a higher proportion of enteral feeds was possible. Four (50%) patients responded to KD treatment within 7 days. During follow-up (median 4.2 months, IQR 1.6–12.3), 5/8 patients—three of them responders—died within 3–12 months after SRSE.

**Conclusions:** In eight patients with SRSE due to severe etiologies including Alpers syndrome, we report an initial 50% response to KD. KD was used early in SRSE and sufficient levels of ketosis were reached early in most patients. Higher ketosis was achieved with combined enteral and parenteral feedings.

## Introduction

Status epilepticus (SE) is one of the most common severe conditions seen at pediatric hospitals. The incidence of SE in childhood is between 17 and 23/100,000, with higher rates in early infancy (1). Hospital mortality is 3%; ~12% of SE patients have suffered from previous epilepsy. Between 10 and 40% of patients progress to refractory SE (RSE) (2–4).

Up to 7–17% of RSE patients progress to super-refractory status epilepticus (SRSE), necessitating treatment with anesthetics at a pediatric intensive care unit (PICU) (5–9). SRSE is defined as SE that continues or recurs 24 h or more after the initiation of anesthetic treatment (10). SRSE is a catastrophic condition with mortality rates of up to 50% (11), and up to 55% of survivors suffering from irreversible severe neurological deficits (12).

The drugs most commonly used in SRSE are midazolam, pentobarbital, ketamine, and inhaled anesthetics. However, to date there is still lack of evidence on the optimal treatment for the individual pediatric patient (10, 13, 14).

The ketogenic diet (KD) is a high-fat, low-carbohydrate diet that mimics the fasting state and is used for the therapy of drug-resistant childhood epilepsy (10, 15–17). Multiple mechanisms of action of KD have been reported. It appears to influence the relative balance between inhibitory and excitatory neurotransmitters and may modify inflammatory processes in the brain (18–21).

Recently, several pediatric case series on KD in refractory (RSE) and SRSE have reported successful interruption in 20–90% within 1–7 days after initiation of KD (22–26), also highlighted in recent reviews (27). Only in adults, a prospective multicenter study provides Class IV evidence of the effectiveness of the KD (28). In most reported pediatric cases, KD was performed via enteral route and ketosis measured only in urine. Furthermore, the majority of reported patients had primarily inflammatory causes of SE such as febrile infection-related epilepsy syndrome (FIRES) or unknown causes (29). Few studies provided detailed descriptions of etiology, in particular results of genetic testing, and electrophysiological data.

The aim of the current study was to evaluate both feasibility and effectiveness of KD in children with SRSE treated at a tertiary care center.

## Materials and Methods

### Study Design

Clinical and EEG data of consecutive children with SRSE treated with KD in addition to standard treatment at the PICU of our center (Department of Pediatrics and Adolescent Medicine, Medical University of Vienna, Austria) from September 2008 to December 2019 were analyzed retrospectively. Data were derived from a longitudinal electronic database containing the complete medical records of all patients with SE at our institution and those treated with KD. For data entry into the database, informed parental consent was obtained. Data collection and analysis were approved by the Ethics Review Board of the Medical University of Vienna (Ethikkommission der Medizinischen Universität Wien, EK.-No. 1594/2019). SRSE was defined as SE that continues or recurs 24 h or more after the initiation of anesthetic treatment (10).

In our center, we adhere to the SE protocol published by Trinka et al. (14) and Trinka and Kälviäinen (30). This staged treatment protocol includes lorazepam, diazepam, clonazepam, or phenobarbital for stage I; phenytoin, valproate, levetiracetam, or lacosamide for stage II; and propofol, midazolam, thiopental, or pentobarbital for stage III (refractory SE). For SRSE, we adhere to the treatment protocol suggested by Shorvon and Ferlisi (10), which recommends general anesthesia including ketamine and AED; however, we use the KD early.

### Study Population and Introduction of Ketogenic Diet

Study inclusion criteria were treatment with KD for SRSE at our PICU and age <18 years at time of presentation. Patients with previous use of KD or contraindications to KD were excluded.

Before initiation of the KD, a thorough neurologic and pediatric examination, video-electroencephalogram (video-EEG), and fasting blood samples were obtained. Furthermore, metabolic diseases contraindicating KD were excluded by reviewing newborn metabolic testing performed at our clinic and by standard metabolic analysis including organic acids in urine (available within 48 h) (31, 32).

The following patient characteristics were documented and used for analysis: the presence of known or unknown etiology including etiology groups according to ILAE criteria (structural, genetic, metabolic, infectious, and immune etiologies) (33); demographic and diagnostic data [age, gender, number of AEDs (before, during, and after KD), epilepsy syndrome, seizure semiology, EEG patterns, and outcome].

After informed consent, an individualized KD diet plan was established for each patient by a trained and experienced dietitian using a computer-based algorithm (34). In most patients, the diet was started at a fat/non-fat ratio of 1:1. Depending on the clinical condition and the neurological state of the patient the diet was administered via parenteral, enteral, or combined parenteral and enteral route. For enteral route, a ketogenic formula (Ketocal; Nutricia Metabolics, Erlangen, Germany; fat/non-fat ratio of 3:1 or 4:1) was used. For parenteral administration, a mixed infusion was prescribed by the ketogenic diet team (pediatric epileptologist A.D. and a dietitian P.T.S.) (34, 35). All other medications were changed to carbohydrate-free formulations when feasible compensating for carbohydrates by adding additional grams of fat (36).

The amounts of ketogenic formula and/or infusion were then increased stepwise to reach a fat/non-fat ratio of 4:1 (maximum), adjusted and monitored by serum beta-hydroxybutyrate (BHB) levels. Our aim was to achieve ketosis (BHB >2 mmol/L) as soon as possible without causing hypoglycemia (serum glucose <50 mg/dl) or severe acidosis (serum bicarbonate >15 mmol/L). When BHB levels were lower than 2 mmol/L, fat/non-fat ratio was increased individually on a daily basis until stable ketosis (between 2 and 5 mmol/L) was achieved. Serum glucose, BHB, and urine ketone levels were measured three times a day while KD was established. Response to KD at the PICU was evaluated daily by a pediatric neurologist and by regular EEG recordings. All EEG recordings were reanalyzed for the purpose of this study. An example of a detailed individual prescription of a KD regimen is given in Table 1.

**Table 1:** Example for the prescription of a ketogenic parenteral nutrition combined with enteral feedings.

Weight (kg)	11	Length (cm)	77	Body surface (m^2^)	0.49
Fluids/m^2^ (ml)		1,500		Fluids/day (ml)	798
Glucose 5%	5 %	350	ml (17.5 g CH)		
Primene^®^ 10% (g/kg/d)	2 g	165	ml (16.5 g Prot)		
SMOF Lipid^®^ 20% (g/kg/d)	3 g	165	ml (33 g Fat)		
Sodium (mmol/kg/d)	2	22	ml		
Potassium (mmol/kg/d)	1	11	ml	**Calories**	
Ca-Gluconate (mmol/kg/d)	1	6	ml	Glucose	74
Glucose-1-phosphate (mmol/kg/d)	1	6	ml (0.36 g CH)	Primene	66
Mg-gluconate (mmol/kg/d)	1	6	ml	SMOF	277
Soluvit^®^ (1 ml/kg/d)		10	ml	Total	417
Vitalipid^®^ (3 ml/kg/d)		3	ml (0.45 g fat)	Calories/kg	38
Peditrace^®^ (1 ml/kg/d)		4^*^	ml		
			**Fat/Non-fat Ratio 0.97: 1**		
		Total:		Infusion rate	
24 h PN without lipids		580	Continuously	24 ml/h	
24 h Lipids, vitamins and trace elements		168	Continuously	7 ml/h	
Hours	24				

*This table contains our established data sheet (34) for prescribing a parenteral KD according to the ESPGHAN guidelines for parenteral nutrition in childhood (35). This includes an age-appropriate daily intake of proteins (in this case 2 g/kg bodyweight) and a maximum daily amount of fats of 3 g/kg bodyweight. Combined enteral KD consisted of a 3:1 fat/non-fat ratio with 6 × 5 ml/days ketogenic formula. Hence, parenteral intake was 96% and enteral intake was 4% of the total energy amount. A maximum fat/non-fat ratio of 0.97:1, and 3:1 was achievable for this child during KD introduction. Nevertheless, with this low fat/non-fat ratio clinically relevant ketosis >2 mmol/l was achieved in 24 h. The child suffered from Alpers Syndrome based on a POLG-mutation (patient five)*.

* *Peditrace® (to a maximum of 4 ml)*.

### Outcome Variables

Primary outcome parameter was frequency (in %) of patients showing clinical and electroencephalographic (EEG) resolution of SRSE within 7 days after starting KD (“responders”). Response was defined as the absence of clinical seizures and ≥50% of the EEG record time showing suppression below 10 μV on longitudinal bipolar montage according to the previous publication by Arya et al. (23).

Secondary outcome parameters included time from initiation of KD to termination of SRSE (in hours), time to achieve clinically relevant ketosis in serum (BHB >2 mmol/L, in hours), time until complete withdrawal of anesthetic drugs after KD start, duration of KD, seizure frequency at last follow-up, and survival (long-term outcome). Moreover, adverse effects were documented in parental diaries.

### Follow-Up Visits

After discharge, urine ketone levels, seizures, nutrition, and adverse effects were documented in a patient diary by parents or caregivers. At each outpatient visit, a thorough pediatric and neurological examination as well as nutritional consults were performed. In addition, blood was analyzed at each outpatient visit including fasting serum glucose and BHB according to our center's standardized protocol (37, 38). Outpatient visits were performed 1, 3, 6, and 12 months after KD start in all patients, also after discontinuing the KD.

### Data Analysis

Patient demographics and clinical characteristics and outcomes are summarized by descriptive statistics (frequency, median, interquartile range (IQR), range). Data analysis was performed using the IBM Statistical Package for Social Science (SPSS Statistics version 26).

## Results

### Patient Demographics

Between September 2008 and December 2019, a total of 98 patients with SE were treated at our center. Eight of these (8.1%) developed SRSE who all received KD in addition to standard of care. Age at onset of SRSE patients was median 13.6 months (IQR 0.9–105). Five patients had epilepsy before SRSE presentation. The etiology was unknown in 2 (25%) patients (Otahara syndrome), and known etiology was observed as follows: genetic etiology in 4 (50%) patients, structural etiology in 1 (12.5%) patient [tuberous sclerosis complex (TSC) and focal cortical dysplasia (FCD) IIB], and autoimmune etiology in 1 (12.5%) patient (FIRES). Genetic testing revealed Alpers syndrome with POLG mutation in 3 (37.5%) patients and a SCN2A-mutation in 1 (12.5%) patient. Seizure types were documented and included spasms, myoclonic seizures, complex partial seizures (including tonic seizures), and generalized tonic clonic seizures. The number of seizure types before KD start was median 3 (mean 2.8, minimum 2–maximum 3).

Number of AEDs before SRSE was median 4 (minimum 0–maximum 7), and number of AEDs at KD start was median 4 (minimum 0–maximum 5).

Demographic data, etiology, epilepsy syndromes, adverse effects, and response to KD are displayed in Table 2. Clinical and EEG outcomes are depicted in Table 3.

**Table 2:** Patients characteristics and KD administration.

**ID/Gender**	**Age at SE onset (months)**	**Treatment response**	**Etiology (ILAE)**	**Diagnosis**	**Epilepsy syndrome**	**AED at KD start**	**Anesthetic drugs before KD**	**Duration of SRSE until KD (days)**	**Time until SE remission after KD start (days)**	**Administration**	Highest Ketosis in mmol/l^*^	**Time until clinically relevant ketosis (hours)**	**Adverse effects**
01/M	0.4	No	Unknown	Unknown	Otahara	0^°^	MDZ	2	2	Enteral (tube)	3.4	56	Dehydration
02/F	1.5	Yes	Unknown	Unknown	Otahara	0^°^	MDZ	9	1	i.v.	4.6	24	Dystrophia, constipation
03/F	37.0	Yes	Genetic	Alpers syndrome	PME	CLB, LEV, MDZ, TPM,	MDZ	4	5	Enteral	5.9	27	High ketosis, diarrhea
04/M	128.2	No	Genetic	Alpers syndrome	PME	LCS, LEV, PB, TPM	PB	42	15	First i.v.—second enteral (tube)	0.9	431	Weight loss, paralytic ileus
05/M	0.7	Yes	Genetic	SCN2A-mutation	IS	LEV, MDZ, PB, VGB,	MDZ, PB	1	1	Enteral (tube)	2	174	Flatulence, constipation
06/F	14.1	No	Structural	TSC, FCD IIB	CPS	LCS, TPM	Propofol^**^, Thiopental	8	33^§^	First i.v.—second enteral	1.3	80	Reduced drinking, diarrhea
07/F	13.0	Yes^+^	Genetic	Alpers syndrome	PME/EPC	LCS, LEV, MDZ, PB,	Ketamine, Propofol^**^	1	1	Combined i.v. and enteral	4.3	28	Hypertriglyceridemia, hyperlipasemia
08/M	147.4	Non	Immune	FIRES	FIRES	LCS, LEV, MDZ, PB, PHT	Ketamine, Propofol^**^	9	15	Combined i.v. and enteral	1.0	236	Pancreatitis, catecholamines, hepatopathy, hypercholesterinemia, hypertriglyceridemia

*ID, identification; M, male; F, female; SE, status epilepticus; ILAE, International League against Epilepsy; SCN2A, sodium voltage-gated channel alpha subunit 2; TSC, tuberous sclerosis complex; FCD II B, focal cortical dysplasia type 2 B; FIRES, febrile infection-related epilepsy syndrome; PME, progressive myoclonus epilepsy; IS, infantile spasms; CPS, complex partial seizures; EPC, epilepsia partialis continua; AED, antiepileptic drugs; KD, ketogenic diet; CLB, Clobazam; LEV, Levetiracetam; MDZ, Midazolam; TPM, Topiramate; LCS, Lacosamide; PB, Phenobarbital; VGB, Vigabatrin*.

+ *Responder within 1st week, relapse of SE after KD discontinuation (adverse effects)*,

° *no AEDs due to suspected GLUT1-deficiency. Pretreatment with pyridoxalphosphate and calciumfolinate*;

§ *until surgery*;

* *during first week*.

*KD was administered orally, via tube or parenterally but also in combined ways. Higher ketosis was achieved when fat/non-fat ratio could be increased due to a higher percentage of enteral feeds. For example, patient 8 was started with a fat/non-fat ratio of 0.94:1 with combined parenteral/enteral feds of 20 and 80%, respectively. Relevant ketosis was reached on day 10 with a fat/non-fat ratio of 3.5:1 at a combined parenteral/enteral ratio of 14 and 86%, respectively. Corticosteroids were weaned 3 days after KD start. In two patients (patient 4 and 5), ketosis was delayed (431 and 174 h, respectively) due to i.v. medication containing glucose. This was detected and resolved*.

** *Propofol was given as single dose (bolus) before KD start in patient 6, 7, and 8. In patient 7 also once during KD (at day 8) as a single dose of 1 mg/kg body weight and in patient 8, three times as single dose of 1 mg/kg body weight at day 4, day 12, and day 13 after KD start*.

**Table 3:** Effectiveness on seizures and EEG and long-term outcome.

**ID/Gender**	**Clinical remission of SE (1st week of KD)**	**EEG remission of SE (1st week of KD)**	**Burst suppression >50% (1st week of KD)**	**Clinical seizure remission (1st week of KD)**	**EEG seizure remission (1st week of KD)**	**Weaning of anesthetic drugs after KD start (days)**	**Long-term outcome**
01/M	Yes	Yes	No	Yes	No	3	Death 12 months after SE
**02/F**	**Yes**	**Yes**	**Yes**	**Yes**	**Yes**	**1**	**Death 3 months after SE (at home)**
**03/F**	**Yes**	**Yes**	**Yes**	**Yes**	**Yes**	**15**	**Death 4 months after SE**
04/M	Yes	Yes	No	No	No	3	Death 6 months after SE
**05/M**	**Yes**	**Yes**	**Yes**	**Yes**	**Yes**	**27**	**KD discontinuation after 1 year, two seizures/month**
06/F	No	No	No	No	No	37^§^	KD ongoing, seizure free after epilepsy surgery
**07/F**	**Yes**	**Yes**	**Yes**	**Yes**	**Yes**	**7**	**KD discontinuation due to adverse effects after 15 days, Death 3 months after SE**
08/M	No	No	No	No	No	61	KD discontinuation due to adverse effects after 10 days, 5 seizures/day

*ID, identification; M, male; F, female; SE, status epilepticus KD, ketogenic diet; M, male; Initial responders are written in bold (patient 2, 3, 5, and 7)*.

§ *8 days after surgery. Patients with EEG and clinical remission of SE during the 1st week of KD are marked in bold*.

### Primary Outcome

Four (50%, 95% CI 22–78%) patients responded to KD treatment (“responders”) according to our criteria, interruption of SE and burst suppression in the EEG (Table 3). Of these patients, two had a diagnosis of Alpers syndrome (patients 3 and 7), one of SCN2A mutation (patient 5), and one of unknown etiology (patient 2). Of the four non-responding patients, two patients (patient 1—unknown etiology and patient 4—Alpers syndrome) had clinical and EEG remission of SE within the first week of KD, but did not fulfill the burst-suppression (>50%) criteria of “responders.” The 2 other “non-responders” did not fulfill any response criteria (patient 6 and patient 8).

### Secondary Outcomes

Secondary outcome parameters are in part displayed in Table 2 [time from initiation of KD to termination of SRSE, and time to achieve clinically relevant ketosis in serum (BHB > 2 mmol/L)]. Time from SE onset to initiation of KD was median 6 days (mean 9.5 days; IQR 1.3–9). Time until clinically relevant ketosis was median 68.0 h (mean 132; IQR 27.3–220.5). Time until complete withdrawal of anesthetic drugs after KD start was median 11 days (mean 19.3, IQR 11.0–34.5). In five patients (63%), anesthetic drugs were withdrawn within 15 days after KD initiation.

### Anesthetic Drugs

Half of the patients (50%) received midazolam (patients 1, 2, 3, 5), one pentobarbital alone (patient 4), and three propofol (two in combination with ketamine [patients 7 and 8, before and while on KD), one with thiopental (patient 6, before KD)]. Propofol was administered as single doses with a maximum of 1 mg/kg bodyweight. In both children who received propofol and ketamine, the KD had to be interrupted due to adverse effects. Only one child who received ketamine and propofol was a responder (patient 7); 3/4 children treated with midazolam showed response (patients 2, 3, 5).

### Adverse Effects

All 8 (100%) patients showed adverse effects of variable severity ranging from gastrointestinal symptoms (diarrhea, constipation, paralytic ileus) and reduced fluid intake to severe derangement of serum lipids, pancreatitis, and need of catecholamines. The KD was discontinued due to adverse effects in 2 (25%) patients: in one “responder” (patient 7) after 15 days due to hypertriglyceridemia and hyperlipasemia; and in one “non-responder” (patient 8) after 10 days due to pancreatitis, need of catecholamines, hepatopathy, hypercholesterinemia, and hypertriglyceridemia.

### Response to KD

**Figure 1:**
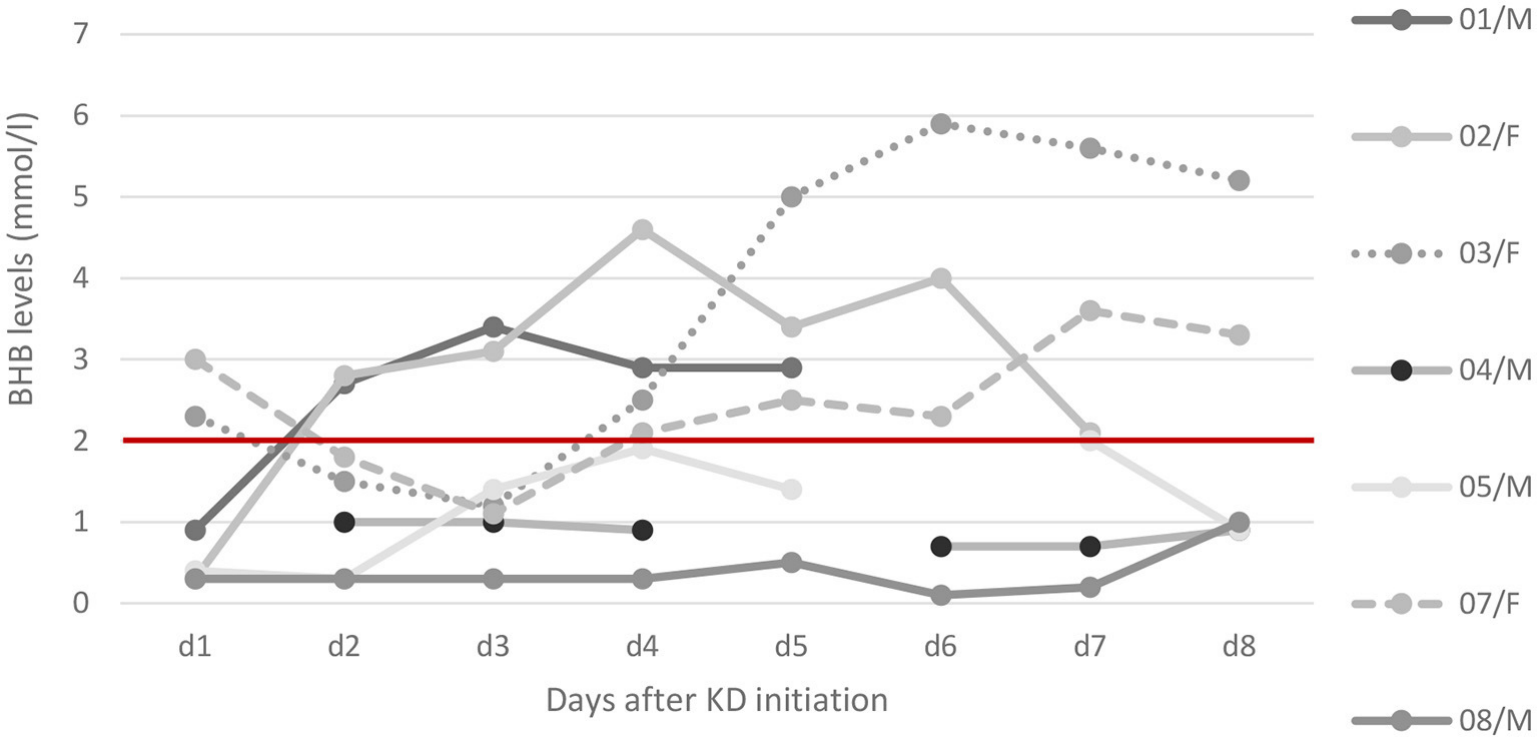
Ketosis after KD initiation. Ketone levels of beta-hydroxybutyrate (BHB) in serum as trajectories from day 1 to day 8. Relevant ketosis (>2 mmol/l) is marked above the red line. Detailed data on ketosis within the first days of KD were available in seven patients: while five patients reached relevant ketosis, the other two (patient 8 and patient 4) showed maximum levels of 0.7 and 0.2 mmol/l, respectively. In patient 8 (FIRES) corticosteroids were administered 3 days before KD start and overlapped for the first 3 days hence delaying ketosis.

Administration and response to KD are depicted in Table 2. Figure 1 displays levels of BHB and shows missing data of BHB levels, as measurements were not feasible at all times at PICU.

Maximum ketosis within the first week was median 2.7 mmol/L (mean 2.9; IQR 1.1–4.5). Fat/non-fat ratio at start of KD was median 1.4:1 (mean 1.7:1; IQR 1:1–2.6:1), and the maximum ratio was median 3:1 (mean 2.9:1, IQR 2.3:1–3.8:1). Total time on KD was median 2.5 months (mean 4.8, IQR 1.1–8.5).

### Survival and Follow-Up

The duration of follow-up after initiation of KD was median 4.2 months (mean 8.4, IQR 1.6–12.3). Three patients survived until last follow up. Patient 5 was a “responder” and currently has two seizures per month. KD was discontinued due to parents' wish after 1 year. However, he has severe encephalopathy due to SCN2A mutation. The other two survivors were “non-responders”: patient 6 became seizure-free after epilepsy surgery 33 days after SE onset and is currently seizure-free on ongoing KD. In patient 8, KD was discontinued due to adverse effects after 10 days. SE was interrupted by anesthetic and AEDs, cortisone, IL-1 receptor antagonist, and vagus nerve stimulator within 4 weeks and burst suppression within the first 6 weeks. He currently has five seizures per day.

Five patients died 3–12 months after SE, three of whom were “responders.” Of the “responders” who died, two patients had Alpers syndrome: one of them died after 4 months (patient 3) and one after 3 months (patient 7) due to cardiopulmonary arrest. The third “responder” with unknown etiology died at home after having been seizure-free for 3 months (patient 2). Of the two “non-responders” who died, SE was interrupted in both patients within the first week with the help of anesthetic drugs: In patient 4 (Alpers syndrome), EEG did not fulfill “responder” criteria; also during follow-up, he showed no changes in EEG and died after 6 months with 10 prolonged seizures a day. The other child (patient 1, unknown etiology) died after 12 months due to infection with ongoing daily seizures (up to 10–20 per day).

## Discussion

We describe a cohort of eight critically ill children with SRSE due to a variety of etiologies. Our incidence of 8% of SRSE among all SE patients lies within the range for pediatric cohorts (5). As a center specialized in KD, we considered this therapeutic option for the treatment of SRSE early. Ketosis could be achieved in most patients within 90 h after KD start (Figure 1). The feasibility of implementing the KD was challenging due to intensive care procedures such as airway management and severe adverse effects such as the occurrence of pancreatitis and the concurrent administration of glucose-containing medications as well as the difficulty to achieve high ketone levels by parenteral routes.

Our retrospective data analysis shows an initial response to KD in 50% of patients, even in etiologies known to be severe and drug resistant (Alpers syndrome, SCN2A mutation). However, 63% of our severely impaired children died in the course of treatment due to the underlying etiology. Only 37% of our patients survived; only one of them was a “responder” to the KD (patient 5/SCNA2 mutation). The combination of KD with midazolam showed better response and fewer adverse effects compared with other anesthetic drugs. In 2/3 patients who received propofol during KD as single doses, the KD had to be discontinued due to adverse effects. In the literature, unfavorable outcome of the combination of propofol and KD has already been described in a single case report (39), when propofol infusion syndrome occurred after introduction of the KD. Therefore, current guidelines for the use of parenteral KD do not recommend the introduction of KD during propofol anesthesia, but if necessary only with caution (40).

In our series of patients, we observed a response rate of 50% (95% CI: 22–78%), which is lower than in some other reports of SE patients treated with KD. This is likely related to severe underlying diseases in our patients. Fifty percent of patients were diagnosed with genetic syndromes, 37.5% of them with mitochondrial DNA depletion syndrome with POLG mutations, a severe degenerative neurological disease with *per se* unfavorable outcome (41). SE interruption was achieved in 2 (of 3) of these patients with Alpers syndrome; however, all three died due to their underlying mitochondrial disease. In spite of thorough diagnostic workup and genetic testing with whole exome sequencing, we could not identify causes of SE in two patients. The highest response rates of up to 90% were reported in cohorts consisting mostly of immune mediated SE such as FIRES (22, 25). Few studies included (or gave detailed information on) genetic causes of SE.

We achieved ketosis within median 3.5 days; in one patient (patient 8) with FIRES high-dose methylprednisolone was administered shortly before KD initiation and overlapping for the first 3 days of KD delaying initial ketosis (cf. Figure 1). Our data are in line with other studies who reported ketosis within a median of 2–5.5 days (22–25), not all of them measuring ketosis in serum.

In 63% of our patients, the KD was administered (at least partly) via parenteral routes. This is a higher proportion compared with other recent studies [10–23% in literature (22, 23)], likely related to more severe general conditions and critical illness. Several studies propose parenteral KD as a feasible method in the intensive care setting (34, 40). Although gastrointestinal motility is frequently reduced in SE, it is important to administer the KD via the enteral route (oral or via gastric tube) as early as possible. The time until relevant ketosis is reached may be delayed due to the limited fat/non-fat ratio if primarily administered parenterally. As soon as an (additional) enteral route is available, ketosis can be achieved more rapidly.

Recent KD studies showed different definitions of adequate ketosis. The most commonly applied biomarker is dipstick ketone testing in urine, although it correlates only poorly with serum ketones. Even the current gold standard in ketosis analysis (31, 36), the measuring of BHB levels in serum is merely a surrogate marker that gives limited information on the metabolic milieu in the brain. In this study, we strictly defined ketosis by serum BHB levels >2 mmol/L (34).

Another area of variability is the definition of response in SE in the literature. We used a strict definition (absence of seizures and suppression-burst ratio ≥50% in EEG within 7 days), as proposed by Arya et al. (23), to allow direct comparison of outcome data.

Although KD has been proposed as effective therapy also for the early (or even prophylactic) treatment of SE (42), at our institution the use of KD was limited to the more severe and super-refractory cases. Nevertheless, the KD was introduced within 9 days after SE onset (median 6 days) in seven of our eight patients. In comparable other studies, this period was significantly longer [median ranging from 12.8 to 18 days (5, 22–34, 36–40, 40–43)]. All our patients (100%) showed a certain degree of adverse effects to the KD; however, the diet had to be discontinued due to adverse effects in only 2 (25%) patients. Although the overall rate of adverse effects is higher than in other reports (0–33%) (22–24, 43), the proportion of discontinued cases (25%) is in line with literature (0–33%) (23, 24, 43). This again might suggest a variability in the definition and reporting of adverse effects associated with KD.

There are some limitations to this study. First, although data were collected prospectively, analysis of SRSE patients was done retrospectively. We minimized this limitation by ensuring that all pediatric patients with SRSE treated with KD were included. Second, the relatively small number of patients limits the generalization of data due to individual therapeutic decision-making. Third, our series has an overrepresentation of children with severe underlying disease (Alpers syndrome, SCN2A), in half of the patients leading to death or poor neurological long-term outcome. This stands in contrast to the literature in which usually more cases with immune-mediated causes are included. However, we describe an initial response in 66% of our patients with Alpers syndrome. Moreover, regular measurements of BHB levels were more difficult to obtain in the intensive care setting during critical illness, also due to concurrent medications.

Strengths of our study are that it represents a group of SRSE patients in whom the KD was started early, as well as the rapid achievement of ketosis in most patients, which underlines the importance of an experienced ketogenic diet team including physicians and dietitians who can introduce and monitor the diet. Another strength is the detailed clinical and etiological description of our patients.

## Conclusion

We report a cohort of eight pediatric SRSE patients treated with KD at our PICU. According to a strict definition of favorable response, 50% responded to treatment. Our data suggest initial effectiveness and safety of early KD, also in patients with severe underlying genetic syndromes. However, only a minority of our severely ill patients survived. In spite of lack of controlled trials in children, our study supports the consideration of KD as treatment option in all patients treated for SE in an intensive care setting.

## Data Availability Statement

The original contributions generated for the study are included in the article/supplementary material, further inquiries can be directed to the corresponding author/s.

## Ethics Statement

The studies involving human participants were reviewed and approved by Ethic Comittee, Medical University Vienna. Written informed consent to participate in this study was provided by the participants' legal guardian/next of kin.

## Author's Note

KD was used early in pediatric SRSE in severely ill children with an initial response of 50%. Ketosis was increased with combined enteral and parenteral feedings.

## Author Contributions

MB: data analysis, paper draft, and revision. CH, SG, PT-S, and JG: acquisition of data and revision. CM: data analysis, data presentation, and critical revision. MF: critical revision. AD: acquisition of data, study design, data analysis, paper draft, and revision. All the authors have read the manuscript, agreed to its submission for publication, approved the acknowledgment of their contributions, and had complete access to the study data.

## Conflict of Interest

AD has received travel reimbursement and speaker honoraria from SHS, Nutricia and Vitaflo. PT-S has received travel reimbursement from SHS, Nutricia and Vitaflo. MF received travel reimbursement from SHS, Nutricia. The remaining authors declare that the research was conducted in the absence of any commercial or financial relationships that could be construed as a potential conflict of interest.

## Abbreviations

KD: ketogenic diet
AED: antiepileptic drug.
